# Facing the urban–rural gap in patients with chronic kidney disease: Evidence from inpatients with urban or rural medical insurance in central China

**DOI:** 10.1371/journal.pone.0209259

**Published:** 2018-12-31

**Authors:** Rui Min, He Wang, Xiaoyan Zhang, Xia Li, Pengqian Fang, Xue Bai

**Affiliations:** 1 Tongji Medical College, Huazhong University of Science & Technology, Wuhan City, Hubei Province, China; 2 College of Politics & Law and Public Administration, Hubei University, Wuhan City, Hubei Province, China; 3 Taihe Hospital of Shiyan City, Shiyan City, Hubei Province, China; Istituto Di Ricerche Farmacologiche Mario Negri, ITALY

## Abstract

**Background:**

In view of the irreversible pathology of progressive exacerbation, the societal burden of chronic kidney disease (CKD) is increasing along with the rise in total health expenditure. Meanwhile, disparities remain among urban and rural citizens with different types of health insurance. This study aimed to assess the socioeconomic disparities between hospitalized CKD patients in urban and rural areas.

**Method:**

A total of 501 CKD inpatients with urban or rural medical insurance (UMI or RMI, respectively) were selected from the top six tertiary hospitals in Wuhan. Demographic and socioeconomic data were collected as influencing factors. Data evaluation was performed using univariate and multivariate analyses.

**Result:**

Socioeconomic characteristics showed differences among hospitalized CKD patients with different health insurances. Patients with RMI were younger, and reported lower education levels, poor domestic economic conditions, shorter duration, and less frequent hospital stays than those with UMI (*P*<0.05). The predictors revealed varying associations between UMI and RMI. Among the hospitalized CKD patients with UMI, male and low-education individuals presented high hospitalization expenses (*β*_*gender*_ = -0.406, *β*_*education level*_ = 0.357, *P*<0.05). By contrast, no significant difference in this aspect was found among RMI inpatients.

**Conclusions:**

Care delivery and reimbursement models should be re-designed and implemented to improve equity among different CKD patients. The national health education should also be enhanced to prevent CKD and provide early treatment.

## Introduction

Chronic kidney disease (CKD) is a general term for heterogeneous disorders affecting kidney structure and function[1]. CKD is defined based on the presence of abnormalities in the structure and function of the kidney for over 3 months, resulting in health complications; CKD is also classified based on the cause, glomerular filtration rate category, and albuminuria category; once progression reaches the final stage, which is known as the end-stage renal disease (ESRD), CKD can only be treated by dialysis and transplantation [2–5] (**S1 Table**).

As an important, chronic, non-communicable epidemic disease with an irreversible pathology of progressive exacerbation that affects people worldwide [6], CKD has become a global health issue. Studies from Europe, Australia, and Asia have confirmed the high prevalence of CKD [7–10]. In China, 130 million Chinese suffer from CKD at a morbidity rate of 10.8%, similar to that in developed countries[11]. Other research reported the globally increasing number of ESRD patients [12–13]; the progressive nature of chronic kidney failure has been causing a substantial burden on global healthcare resources [14]. In the United States, the annual Medicare fee-for-service spending for the beneficiaries of patients with ESRD rose to US$33.9 billion in 2015, whereas the per person-per year spending totaled $88,750 for hemodialysis and $75,140 for peritoneal dialysis [15–16].

As a chronic disease, CKD requires long-term medical care. Patients with CKD face a higher risk of dying from complications, such as cerebrovascular disease, than from kidney failure [17]. This result can lead to considerable medical expenses not only for treating nephrological diseases, but also for treating cerebrovascular diseases. The cost of nutritional support is also important. A report showed that 10% of the world’s population is affected by CKD, and millions of people die each year given the lack of access to affordable treatment [18]. Given a preliminary estimated rate, about 100 million adults in China suffer from CKD. The societal burden of CKD increases along with the rise in total health expenditure. One important issue is that poverty is constantly related to diseases, that is, poverty may cause diseases, and diseases also result in poverty. As mentioned previously, CKD treatment is expensive. Furthermore, all treatments can only retard the development rather than cure ESRD. Except for the physical discomfort caused by CKD, patients and their families experience substantial economic and psychological pressure. Hence, the Chinese government is seeking for rational strategies to constrain the cost while maintaining satisfactory standards of patient care. Since the launching of the New Health Reform in China in 2009, the universal coverage of basic health insurance has gained remarkable achievements. Currently, three major basic medical insurance systems are available in China: the New Cooperative Medical Scheme for 275 million rural residents (NCMS or Rural Medical Insurance, RMI), the Urban Employees’ Medical Insurance, and the Urban Residents Medical Insurance for urban citizens. The latter two are collectively known as Urban Medical Insurance (UMI), which covers 748 million urban citizens. Medical security aims to achieve health equity among different groups of citizens via universal coverage of healthcare insurance. People with health insurance face a low risk of becoming impoverished. However, healthcare insurance inequity cannot be ignored. Healthcare insurance inequity; not only covers the inequity of expenses but also the unfairness of the policy-design level. The inherent defects of system design, such as the differences in funding source, financing level, and reimbursement rate (**S2 Table**)[19–20], have impeded healthcare insurance equity. Meanwhile, the inequity in healthcare insurance may interfere with the choice of treatment and compliance of the insured population[21]. Understanding the dynamic factors contributing to health disparities and designing effective interventions embedded in healthcare systems have been a challenge for academicians and policy makers. The first and most important step for improving health security level is to distinguish the population health demands in different insurance groups, and address the inequity of health insurance between urban and rural residents. Literature review showed that CKD causes massive economic and social burden worldwide, and limited research reports originated from China. Moreover, previous research on CKD rarely focused on the inequity of healthcare insurance. Therefore, this research aimed to explore the health insurance inequity between urban and rural residents by illustrating the disparities among CKD patients with UMI and RMI.

## Methods

A cross-sectional population-based survey was conducted in tertiary-level hospitals in Wuhan, Central China. A multistage, stratified sampling method was used to obtain a representative sample of people aged 18 years or older from the general population. This investigation obtained the approval of the Ethics Committee of Tongji Medical College, Huazhong University of Science and Technology (**S1 File**).

### Participants

A total of 540 hospitalized CKD patients (90 inpatients per hospital) visiting the top six tertiary level hospitals in Wuhan from Oct. 1, 2015 to Dec. 31, 2015 participated in the study. The top six tertiary hospitals were selected on the basis of the “Top 10 Famous Hospitals in the Wuhan Area” ranked by the Wuhan Health Commission. All hospitals were general hospitals and featured an independent clinical department of nephrology (**S3 Table** provides the details of the six hospitals; **S1 Fig** proves the sample size formula). According to the annual report data, 65.34 million inpatients obtained their medical care in the six sample hospitals. The morbidity of CKD in China was reported to reach 10.8%. In this study, the population of hospitalized CKD patients was estimated at 7.06 million, and the enrolment rate of healthcare insurance was 95% on average. The study sample was estimated at 540 patients. All participants were adult (≥18 years) with clear consciousness, and gave verbal permission to use their information.

### Questionnaire

A questionnaire was for hospitalized CKD patients designed in three parts: sociodemographic data, attitude for health insurance, and quality of life appraisal. The questionnaire was formulated through the Delphi consultation method, and completed by hospital managers, doctors from the department of nephrology, patients with CKD, and professors of healthcare services (**S2 File**). A pilot study was conducted in Wuhan Tongji Hospital to ensure that the questionnaire was intuitive, understandable, and flexible. Personal identifying information were removed and retained anonymously during the entire study process. A convenience sampling method was used to select the interviewees for the patient questionnaire. As the participants needed to provide recall information, and their feelings must be considered, all interviews were completed by students with professional interviewing skills from Tongji Medical College with assistance by health staff.

### Data collection

Data were obtained from the patient questionnaires and official statistical reports. The demographic and socioeconomic data included age, gender, personal/household income (divided into five levels in accordance with the National Statistical Bureau and the World Bank; for a typical Chinese family of three, an annual household income under $1,200 was defined as poverty, $1,200 to $3,000 was low income, $3,000 to $12,000 was lower middle income, $12,000 to $37,000 was upper middle income, and over $37,000 was high income), family consumption (expenditure incurred by resident family on goods or services and includes the shares of expenses on health, education, food, or housing), and educational level (low educational level was defined as less than senior middle school; high educational level was defined as accomplishment of undergraduate course or higher). Catastrophic health expenditure (CHE) was defined as an out-of-pocket payment for healthcare ≥ 40% of an annual family consumption following the definition of World Health Organization [22]. Meanwhile, the average consumption of inpatients (hospitalization expenses), mode of payment, function test, stage of CKD (ESRD that requires patients to undergo dialysis, and early-stage are patients in G1 to G4 stage), medical history, etiology of CKD, and body mass index (BMI) were also included in the questionnaire.

### Measures of medical expenditure

Health consumption is an important component of family consumption. Medical expenditure can reflect the financial burden of patient diseases and utilization of healthcare services[23–25]. Medical expenditure comprises direct and indirect health costs. Direct cost includes costs of medicine, examinations, consultations, treatments, inpatient stays, and other direct healthcare services. Medicine cost, inspection cost, and nursing cost are the main paid items under hospitalization expenses in China. The medicine cost denotes the expenses on drugs used in patient treatments. The inspection cost denotes the expenses on laboratory evaluation or auxiliary examinations. The nursing cost includes the expenses on nursing care. Indirect costs cover transportation, special diets, and family company. Medical expenditure has been reported to be influenced by various factors(gender, age, educational level, health insurance coverage, personal health status, seriousness of illness, and health service capability of the hospital)[23, 25–28].

### Statistical analyses

EpiData3.0 (EpiData Association, Odense, Denmark) was used for data entry, and SPSS20.0 (IBM Analytics, Armonk, NY, USA) was utilized for statistical analysis. Univariate and bivariate statistical models were used. Continuous variables were expressed as mean ± standard error (S.E.) for normally distributed data or as median and frequency (%) for non-normally distributed data. T-test was adopted for normally distributed continuous data. Categorical data were presented as proportions and analyzed with Chi-square test. A two-sided test with *P* < 0.05 was considered statistically significant. Meanwhile, linear regression analysis was used to calculate the connection between health expenditure and potential influencing factors.

## Results

### Population characteristics

A total of 540 inpatients participated in this study, and gave a high response rate of 92.8% (**S2 Fig**). Nine patients could not finish the investigation as they refused to provide certain information. Among the remaining 531 respondents, 268 (50.5%) were females. A total of 316 patients (59.5%) had UMI. Thirty patients with civil servant medical care subsidies were excluded because of the uniqueness of the payment system and the difference of this system from UMI. The average BMI value was 21.86±3.35 (21.93 for UMI patients and 21.75 for RMI patients). Patients with RMI were younger (*P* < 0.001) and attained a lower education level (*P* < 0.001) than those with UMI (Table 1).

**Table 1:** Demographic characteristics and basic status of CKD inpatients.

Characteristics	Total(n = 531)	Medical insurance type		
		UMI (n = 316)	RMI (n = 185)	*P* value
** Age**				**< 0.001**
≤24	35(6.6%)	12(3.8%)	23(12.4%)	
25–34	51(9.6%)	25(7.9%)	24(13.0%)	
35–44	47(8.9%)	26(8.2%)	20(10.8%)	
45–54	118(22.2%)	63(19.9%)	52(28.1%)	
55–64	113(21.3%)	72(22.8%)	34(18.4%)	
≥65	167(31.5%)	118(37.3%)	32(17.3%)	
** Gender**				**0.929**
Male	263(49.5%)	156(49.5%)	91(49.2%)	
Female	268(50.5%)	160(50.5%)	94(50.8%)	
** Married**	413(77.9%)	248(78.7%)	140(75.7%)	**0.005**
** BMI level**				0.568
Underweight	87(16.4%)	50(16.3%)	31(16.8%)	
Normal	302(56.9%)	186(59.4%)	104(56.5%)	
Overweight	109(20.5%)	61(19.5%)	39(21.2%)	
Obesity	29(5.5%)	16(5.1%)	10(5.4%)	
** Education level**				**< 0.001**
High	158(29.8%)	110(34.8%)	12(7.41%)	
Low	373(70.2%)	206(65.2%)	150((85.4%)	
** Occupational status**				**0.020**
Employment	164(30.9%)	88(27.8%)	70(37.8%)	
Unemployment	367(69.1%)	228(72.2%)	115(62.2%)	

### Economic characteristics

The annual personal income was $7,515.00 before illness (before diagnosis with CKD) and $6,251.26 after illness (after diagnosis with CKD). The average annual family consumption was $8,122.81, and the medical expenditure was $3,409.26 (42.0% of family consumption). A total of 222 patients suffered from CHE. The average cost for each hospitalization reached $2,298.95, of which 31.9% accounted for the medicine cost and 28.1% covered the inspection cost (Table 2).

**Table 2:** Changing of economic characteristics in UMI/RMI patients.

Characteristics		Total		Medical insurance type			
				UMI		RMI	*P value*
**Annual income (**$\bar{X}\pm SE$**)**							
personal	before the illness		7515.00±853.14		8635.26±1143.20	5961.09±1262.04	0.122
	after the illness	6251.26±874.99		6907.86±1190.38		5340.50±1281.39	0.379
household	before the illness		11072.21±3256.40		13315.50±5064.35	7082.64±832.54	0.359
	after the illness	8571.62±478.53		9124.12±574.21		7591.58±848.20	0.124
**Annual family consumption (**$\bar{X}\pm SE$**)**							
** total**		8122.81±313.57		6344.94±370.68		7250.52±573.20	0.522
: health consumption		3409.26±176.82		3367.58±197.15		3479.32±339.68	0.760
**CHE** [n (%)]							
** total**		222(44.3%)		143(45.3%)		79(42.7%)	0.579
**Average cost/ hospitalization (**$\bar{X}\pm SE$**)**							
** total**		2298.95±238.92		2087.79±329.35		2665.57±316.07	0.245
: medicine cost		733.45±70.83		641.98±84.12		902.33±127.21	0.079
inspection cost		645.87±52.13		569.25±58.42		776.39±98.64	0.055
nursing cost		176.12±26.08		156.55±34.45		214.19±37.36	0.297

**Fig 1:**
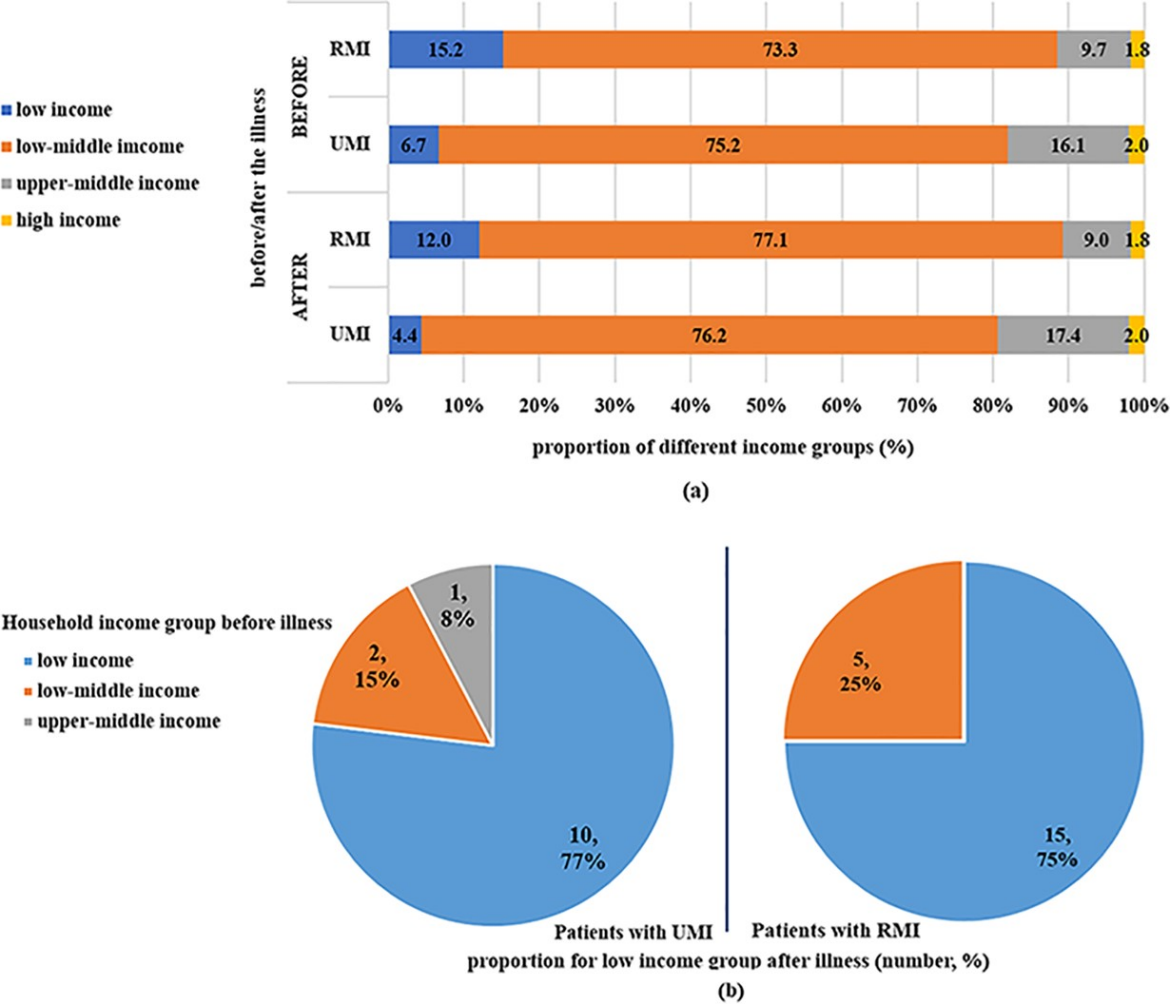
a) Changing among 4 different household income groups with UMI/RMI. b) Pie chart for composition of patients with low household income after illness with UMI/RMI.

In general, patients with CKD reported low household incomes. Among the groups with different household income levels, patients in low income and low middle-income level accounted for 81.9% of those with UMI before illness. This percentage was 6.6% lower than that of patients with RMI (*P*< 0.001). The gap between urban and rural residents in low income and low middle-income level changed to 8.5% after illness (80.6% for UMI patients versus. 89.1% for RMI patients, *P* < 0.001) (Fig 1A). Furthermore, 27 UMI patients and 19 RMI patients reported changes in household income after illness. The pie chart explains the composition of patients with low household incomes after the illness: for the UMI patients, the proportion of patients transferred from their original income group to the low-income group reached 15.4% and 7.7% for the low-middle and upper-middle income populations, respectively; for the RMI patients, 25% of the low-middle income population transferred to the low-income group (Fig 1B).

### Basic status and life quality

A total of 185 patients were with ESRD and thus required dialysis. Compared with rural patients, urban patients reported a longer duration of illness (*P* = 0.001), more frequent hospital stays (*P* = 0.043), and poorer health status (*P* = 0.049) (Table 3).

**Table 3:** Basic status and life quality of CKD inpatients with UMI/RMI.

Characteristics	Total	Medical insurance type		
		UMI	RMI	*P* value
Duration of illness time(years)*				**0.001**
Under 5 years	303(57.1%)	152(63.6%)	133(82.1%)	
5–10 years	59(11.1%)	44(18.4%)	12(7.4%)	
Over 10 years	66(12.4%)	43(18.0%)	17(10.5%)	
Frequency of hospital stay/month**				**0.043**
once	285(56.9%)	171(54.1%)	114(61.6%)	
twice	104(20.8%)	64(20.3%)	40(21.6%)	
Over 3times	112(22.4%)	81(25.6%)	31(16.8%)	
**Dialysis (**ESRD patients**)**				0.308
Yes	185(36.9%)	122(38.6%)	63(34.1%)	
No	316(63.0%)	194(61.4%)	122(65.9%)	
** Health status**				**0.049**
much better than last year	7(1.4%)	5(1.6%)	2(1.1%)	
better than last year	38(7.6)	24(7.6%)	14(7.6%)	
no change	134(26.7%)	70(22.2%)	64(34.6%)	
worse than last year	172(34.3%)	116(36.7%)	56(30.3%)	
much worse than last year	150(29.9%)	101(32.0%)	49(26.8%)	

*: from the first CKD treatment to study recruitment. 100 patients forgot the time they got their first diagnosis.

**: times of hospitalization in the month which the study recruitment

### Association between medical expenditure and different factors

**Fig 2:**
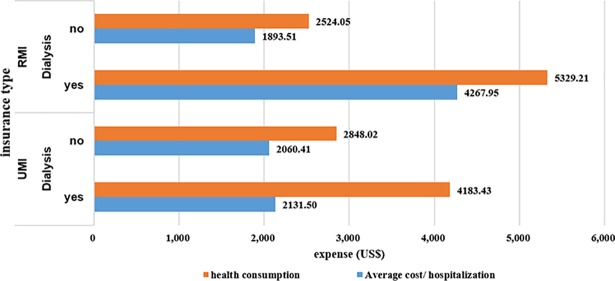
Difference in expense between patients in earlier stage (G1-G4) and Dialysis (ESRD) (classified by insurance type).

No significant difference in hospitalization expenses and health consumption was found between the UMI and RMI patients. Four factors showed an association with medical expenditure among CKD patients. Patients with ESRD exhibited substantial expenses in healthcare (Table 4). Patients with RMI incurred higher expenses in healthcare than patients with UMI (1.03 times in health consumption, and 1.27 times in hospitalization cost.). For patients with RMI, at the 1% significance level, the Chi-square test showed a significant difference in health consumption and hospitalization cost between the ESRD patients and those in early-stages (G1 to G4) (Fig 2). For patients with UMI, a significance difference was observed in the results for hospitalization cost (*P* = 0.005, Fig 2).

**Table 4:** Factors associated with medical expenditure of CKD patients.

	Health consumption		Hospitalization expenses		
	($\bar{X}\pm SE$)	*P* value		($\bar{X}\pm SE$)	*P* value
**Gender**		**0.022**			0.826
Male	3753.28±297.72			2851.18±454.73	
Female	3080.06±191.77			1735.45±118.94	
**Occupational status**		0.159			**0.005**
Employment	3376.59±225.17			2417.21±342.52	
Unemployment	3479.16±276.73			2045.77±159.51	
**Education level**		0.386			**0.039**
High	3449.78±220.04			2199.28±192.35	
Low	3300.91±276.35			2557.77±700.94	
**Medical insurance type**		0.760			0.245
UMI	3367.58±197.15			2087.79±329.35	
RMI	3479.32±339.68			2665.57±316.07	
**Duration of illness time(years)**		**0.012**			**0.001**
Under 5 years	3106.97±234.25			2488.37±231.94	
5–10 years	4525.14±560.50			3679.80±1619.99	
Over 10 years	4175.83±591.33			1396.95±158.69	
**Dialysis (**ESRD patients**)**		**0.000**			**0.004**
Yes	4575.74±385.20			2830.46±340.69	
No	2721.31±153.35			1995.76±320.00	

In regression analysis, illness duration and being a dialysis patient were independently associated with medical expenditure. Males exhibited a higher health consumption than females (Tables 5 and 6).

**Table 5:** Liner regression analysis of health consumption and related factors.

Index	β	SD	B*	t	P	95%confidence interval	
						Lower	Upper
**[constant]**	10.418	0.285		36.593	0.000	9.858	10.978
**Gender(male)**	-0.298	0.100	-0.154	-2.995	0.003	-0.494	-0.102
**Duration of illness time(years)**	-0.347	0.105	-0.170	-3.291	0.001	-0.554	-0.140
**Dialysis (ESRD patients)**	-0.187	0.068	-0.143	-2.768	0.006	-0.320	-0.054

Note: 1) Use ln (health consumption) as the dependent variable

2) *: Standardized coefficients

**Table 6:** Regression analysis of hospitalization expenses and related factors.

Index	β	SD	B*	t	P	95%confidence interval	
						Lower	Upper
[constant]	7.979	0.259		30.790	0.000	7.470	8.489
**Occupational status**	0.072	0.107	0.035	0.676	0.500	-0.138	0.282
**Education level(low education)**	0.111	0.114	0.051	0.974	0.330	-0.113	0.335
**Duration of illness time(years)**	0.187	0.066	0.140	2.831	0.005	0.057	0.317
**Dialysis (ESRD patients)**	-0.187	0.068	-0.143	-2.768	0.006	-0.320	-0.054

Note: 1) Use ln (hospitalization expenses) as the dependent variable

2) *: Standardized coefficients

### Stratified analysis by medical insurance type

**Fig 3:**
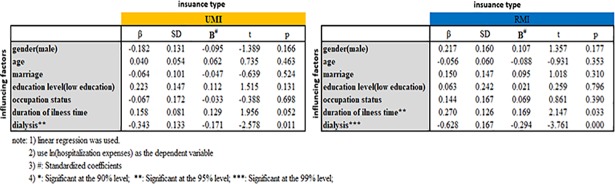
Difference of health consumption and related factors stratified by insurance type.

**Fig 4:**
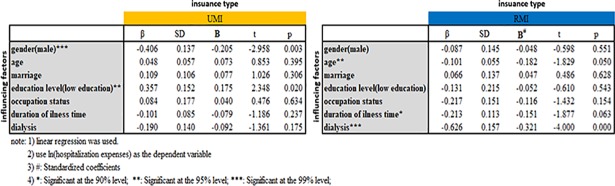
Difference of hospitalization expenses and related factors stratified by insurance type.

In UMI and RMI, long durations and serious health status after being diagnosed with CKD directly affect medical expenditures. Male and low-educational level hospitalized CKD patients with UMI reported high hospitalization expenses (*β*_*gender*_ = −0.406, *β*_*education level*_ = 0.357, *P*<0.05). Among the RMI inpatients, age, duration of illness, and being a dialysis patient were negatively correlated with hospitalization expenses (Figs 3 and 4).

## Discussion

This study conducted a retrospective analysis of the relationship between the medical expenditures for the treatment of CKD and medical insurance type. Data showed that urban citizens with CKD were older, possessed higher level of education, and featured a lower employment rate and higher marriage rate than rural citizens. Family burdens were significantly heavier among the RMI patients than among the UMI patients given the large gap in low-income level after CKD diagnosis. Meanwhile, urban patients suffered longer and experienced poorer health status than rural patients. Besides aiming for an equitable universal coverage of health insurance, authorities and insurance managers should recognize the disparities among different patients with different demographic statuses.

Notably, the UMI patients presented a longer duration of illness and more frequent monthly hospital stays than the RMI patients. These findings were similar to those of other studies in China [21], the United States [29], South America [8, 10], and Australia [10], showing that insurance disparity may affect the choice of treatment of CKD patients. The disparities were attributed more to the design level more than the action level. In general, on the basis of clinical practice guidelines[2, 30–31], the hospitalized CKD patients in top-level hospitals were intractable cases, including complicated patients who cannot be clearly diagnosed and patients with serious complications. Hospitals, especially high-level hospitals, are normally concentrated in developed regions; this trend implies that urban residents possess more timely access to healthcare than rural residents[32]. Convenience is another key factor in delaying the progress of CKD. The distance of RMI patients from top-level hospitals is generally much longer than that of the UMI patients. Several studies have shown that the distance from hospitals is positively related to the duration of illness and mortality among CKD patients; and may result in less contact of nephrologist to patients in remote areas. Other studies have shown similar results [33–35].

Change in household income is one of the direct indicators reflecting the disease burden [36]. The study showed the expanded number of patients with CKD under an income level of $12,000 after illness. For the low-middle income population, illness is a risk factor for poverty, especially for rural residents. Although urban patients presented a longer duration of illness and more frequent hospitals stays than rural patients, most rural residents engage in manual work, and feature a simpler income source, and lower income level [23]. Once suffering from CKD, patient completely or partially lose their labor capacity, resulting in the rapid decline in household income. Another reason is the insurance coverage disparity. Patients with UMI obtained insurance reimbursement for 66.97% of their medical consumption, whereas the rate for RMI patients amounted to 58.5%[20]. That is, rural patients shouldered 8.4% higher out-of-pocket expense than urban patients. Meanwhile, higher the hospital level is, lower the reimbursement rate is. However, further analysis showed no significant difference in hospitalization expenses and health consumption between UMI and RMI patients. A significant difference in expenses was observed among ESRD patients with different insurances. These observations proved that RMI may increase the risk of patients with CKD to move down to the lower income group and that inequity in healthcare insurance had led to a disparity in medical expense.

Socioeconomic disparities exist between different insurances, affecting the medical expenditure for hospitalized CKD patients. Males, who are the main laborers of the family, presented a higher health consumption than females. According to previous reports, this observation may be related to the progression of CKD, which was proven to develop faster in males than females owing to the greater risk factors in the former than in the latter[32]. In the present research, among patients with UMI, the hospitalization expense for males was 2.46 times as much as that for females. For the high-educational level patients, the value was 2.80 times that of low-educational level patients. However, the socioeconomic indicators were not independently associated with the medical expenditures under the RMI group. This finding was similar to that of a study in Brazil [37]. CKD is an irreversibly progressive disease. Thus, the treatment and medicine of CKD are limited in the medical insurance catalog. Although the inpatient reimbursement rates of different insurances varied, no significant difference was found in medical expenditures between the currently hospitalized patients possessing UMI and those with RMI.

The awareness and ability for self-management in health are closely associated with socioeconomic indicators, and they are important factors for disease prevention and treatment. In the previous studies in China, the average education level of rural areas was lower than that of urban areas; hence, given their poorer awareness and self-management ability, rural patients presented a higher prevalence rate of CKD than urban patients[11]. Similarly, the present study showed that rural patients exhibited lower education level and household income than urban patients [38]. In addition, rural patients were younger [39] than the urban patients; this result was related to the lower health literacy and awareness of CKD of the former; such condition can delay diagnosis and treatment.

### Study limitation

A high-quality data collection method and an analysis method were nested in a well-designed population-based study among hospitalized CKD patients. However, this study presented several limitations. First, although the cross-sectional design enabled the estimation of health expenditure of hospitalized CKD patients, the data sample was small and collected only in a large city in Central China. Second, all economic information was based on recall, and recall bias was another influencing factor. Although we employed professional investigators to minimize these biases, such predilections cannot be completely eliminated. Third, as a traditional habit, most people were reluctant to reveal their economic information in addition to their financial details. The economic burden of disease was difficult to evaluate and compare with those in other countries. Fourth, this study differed from other research that used subjective evaluation as a substitute for objective indicators.

## Conclusion

Social and economic disparities were analyzed to be substantial between patients possessing UMI and those with RMI. These disparities are sufficient to alert policy makers to focus on expanding the benefit package and redesigning the health security system. In the integration of UMI and RMI, the key issue is the equity of healthcare insurance. Care delivery and reimbursement models should be re-designed as follows. 1) The targeted intervention for rural patients should be reinforced. 2) The security level for patients with ESRD and low-middle income level patients should be improved, and the Critical Illness Insurance should be allowed to play a full role to prevent poverty caused by illness. 3) Suitable treatments should be implemented on the basis of household economic status, local medical service level, and insurance type of these patients. Raising the health literacy of all citizens to a high level is an effective and convenient way to improve the awareness and self-management ability of citizens for the early prevention and discovery of CKD.

## Supporting information

S1 Table Introduction for CKD stages. Stages of chronic kidney disease, as defined by the Kidney Disease Outcomes Quality Initiative.
(PDF)

S2 Table Basic introduction of UMI and RMI. Differences between UMI and RMI among management, level of pooling, enrolment rate, benefits package, reimbursement and its rate.
(PDF)

S3 Table Introduction of Top 10 famous hospitals in Wuhan. Health service delivery information of “Top 10 famous hospitals in Wuhan area”.
(PDF)

S1 Fig Formula for calculating the sample size. This is an explanation of how we settled down the sample size of this study.
(TIF)

S2 Fig Effective sample size of study. This is an illustration of the effective sample size of this study.
(TIF)

S1 File Ethic approval of this study. (PDF)

S2 File CKD Outpatient Questionnaire Form & Data set. (RAR)

S3 File Checklist. (PDF)

## Abbreviations

BMI: Body mass index
CKD: Chronic kidney disease
ESRD: End-stage renal disease
RMI: Rural Medical Insurance
UMI: Urban Medical Insurance
