# Supplementary material for: Facing the urban–rural gap in patients with chronic kidney disease: Evidence from inpatients with urban or rural medical insurance in central China
Source: PLoS One. 2018 Dec 31;13(12):e0209259. doi: 10.1371/journal.pone.0209259 (PMC6312298; doi:10.1371/journal.pone.0209259)
Supplement: S1 Table — Stages of chronic kidney disease, as defined by the Kidney Disease Outcomes Quality Initiative. (PDF) [file pone.0209259.s001.pdf]

S1 Table. Introduction for CKD stages.

Stages of chronic kidney disease, as defined by the Kidney Disease Outcomes Quality Initiative

**Description**	**GFR (ml / min ·1.73 m2)**	**Stage of CKD**
Normal or high	≥90	G1
Mildly decreased	60-89	G2
Mildly to moderately decreased	45-59	G3a
Moderately to severely decreased	30-44	G3b
Severely decreased	15-29	G4
Kidney failure	<15	G5

GFR: estimating glomerular filtration rate category
