# Supplementary material for: Facing the urban–rural gap in patients with chronic kidney disease: Evidence from inpatients with urban or rural medical insurance in central China
Source: PLoS One. 2018 Dec 31;13(12):e0209259. doi: 10.1371/journal.pone.0209259 (PMC6312298; doi:10.1371/journal.pone.0209259)
Supplement: S2 Table — Differences between UMI and RMI among management, level of pooling, enrolment rate, benefits package, reimbursement and its rate. (PDF) [file pone.0209259.s002.pdf]

S2 Table. Basic introduction of UMI and RMI.

Differences between UMI and RMI among management, level of pooling, enrolment rate, benefits package, reimbursement and its rate.

	**UMI**	**RMI**
**Overseeing ministry**	Ministry of human resource and social security, MoHRSS	National health commission, NHC
**Level of pooling**	Prefecture/municipality	County
**No. of risk pools (approx.)**	330	2,600
**Enrolment rate(%)**	>95 [a]	98.9[b]
**Benefits package**	in- and outpatient care<br>critical and chronic outpatient care	in- and outpatient care<br>critical and chronic outpatient care
**Reimbursement (yuan)**	813.36 billion	289.04 billion
**Mandated reimbursement rate(%)**		
-inpatient care	80% for employee, 70% for residents	>75%
-outpatient care	no	no
**Effective reimbursement rate(%)[c]**		
-inpatient care	66.97	58.5
-outpatient care	50	50

Sources: China Health Statistics Yearbook 2015 (NHC). *Summary of National Social Insurance in 2015* [in Chinese] (MoHRSS). *Statistical Bulletin of the Development of Human Resources and Social Security, 2015* [in Chinese] (MoHRSS). *The New Rural Cooperative Medical Scheme Statistical Manual, 2013* [in Chinese] (NHC). *Policy of the New Rural Cooperative Medical Scheme* (2012-2015) [in Chinese] (NHC). *Policy of healthcare insurance* (2012-2015) [in Chinese] (MoHRSS). "[1]Green book of health reform and development: Annual report 2016, special issue for reform and development in healthcare insurance system [in Chinese] (PQ Fang, et al.).

Note: a. reported in *The 12th five-year report: achievements in employment and social security* (MoHRSS,2015)

b. reported in *China Health Statistics Yearbook 2015* (NHC,2015)

c. reported in *Green book of health reform and development: Annual report 2016*
