# Supplementary material for: Facing the urban–rural gap in patients with chronic kidney disease: Evidence from inpatients with urban or rural medical insurance in central China
Source: PLoS One. 2018 Dec 31;13(12):e0209259. doi: 10.1371/journal.pone.0209259 (PMC6312298; doi:10.1371/journal.pone.0209259)
Supplement: S3 Table — Health service delivery information of “Top 10 famous hospitals in Wuhan area”. (PDF) [file pone.0209259.s003.pdf]

S3 Table. Introduction of Top 10 famous hospitals in Wuhan.

Health service delivery information of “Top 10 famous hospitals in Wuhan area”

Hospital	General hospital/Specialized hospital	Outpatients (10^6)	Inpatients (10^6)	Length of Stay	Rank	Department of nephrology	Sampling
Tongji Hospital of Huazhong University of Science and Technology	General hospital	3.81	15.35	10.28	1	√	√
Union Hospital of Huazhong University of Science and Technology	General hospital	3.51	15.84	10.48	2	√	√
Hubei Provincial People's Hospital	General hospital	1.90	11.10	10.58	3	√	√
The Central Hospital of Wuhan	General hospital	1.28	9.35	10.31	4	√	√
Wuhan No.1 hospital	General hospital	2.28	7.10	10.07	5	√	√
Wuhan Women and Children Medical Care Center	Specialized hospital	1.94	7.37	6.51	6	×	×
Zhongnan Hospital of Wuhan University	General hospital	1.17	6.60	11.5	7	√	√
Wuhan Puai Hospital	General hospital	1.16	6.07	10.89	8	×	×
Hubei Province Maternal and Child Health Hospital	specialized hospital	1.72	5.46	7.52	9	×	×
Hubei Provincial Hospital of Traditional Chinese Medicine	Traditional medicine hospital	1.54	4.17	11.39	10	√	×

Note: this ranking list was published by Wuhan health commission on Sept.12, 2014, six months before the study.
