# Supplementary figures and images for: Facing the urban–rural gap in patients with chronic kidney disease: Evidence from inpatients with urban or rural medical insurance in central China
Source: PLoS One. 2018 Dec 31;13(12):e0209259. doi: 10.1371/journal.pone.0209259 (PMC6312298; doi:10.1371/journal.pone.0209259)

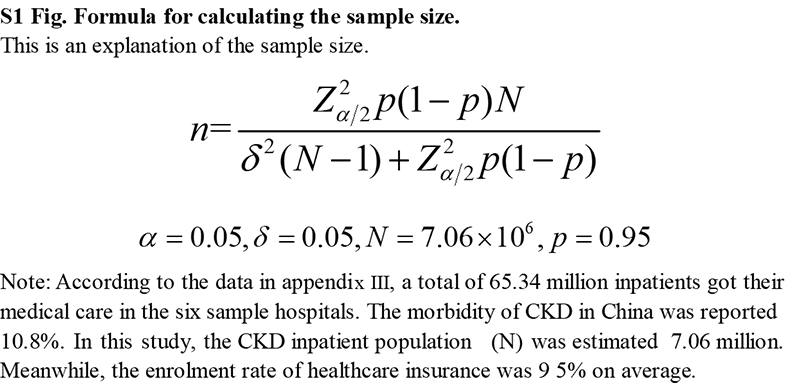

Supplement: S1 Fig — This is an explanation of how we settled down the sample size of this study. (TIF) [file pone.0209259.s004.tif]

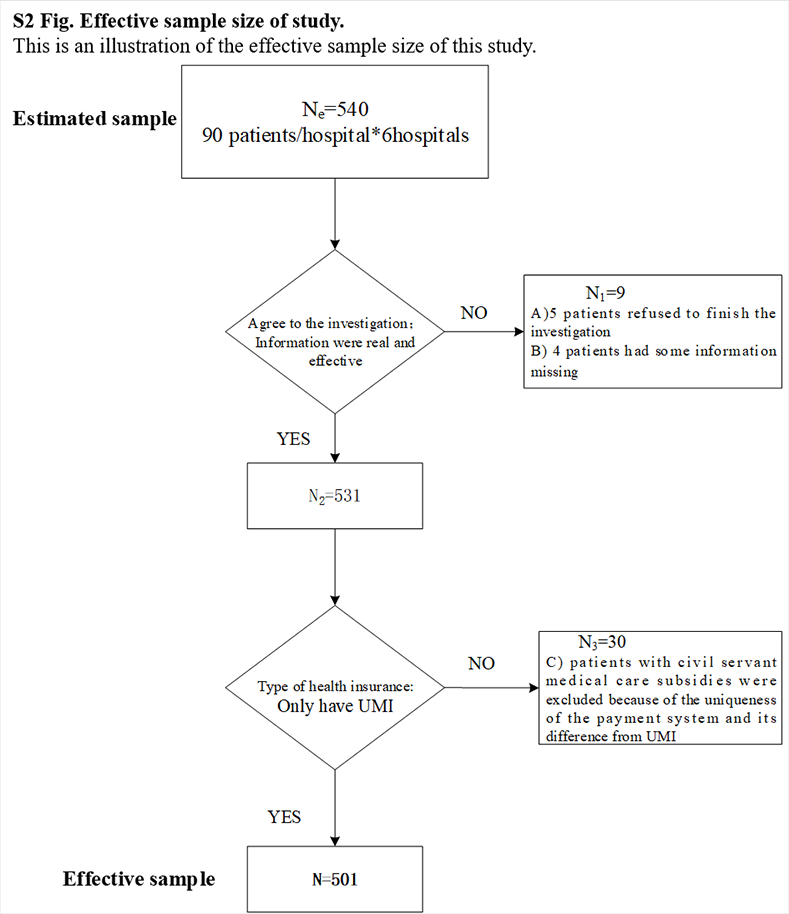

Supplement: S2 Fig — This is an illustration of the effective sample size of this study. (TIF) [file pone.0209259.s005.tif]
