# Supplementary material for: Facing the urban–rural gap in patients with chronic kidney disease: Evidence from inpatients with urban or rural medical insurance in central China
Source: PLoS One. 2018 Dec 31;13(12):e0209259. doi: 10.1371/journal.pone.0209259 (PMC6312298; doi:10.1371/journal.pone.0209259)
Supplement: S1 File — (PDF) [file pone.0209259.s006.pdf]

RESEARCH ETHICS COMMITTEE APPROVAL FORM

The Ethics Committee of Tongji Medical College, Huazhong University of Science and Technology (IORG No: IORG0003571) gave a final APPROVAL on 30/12/2014 for the study Research on medical insurance management of patients with Chronic Kidney Disease in Wuhan which is conducted by Prof. Fang Pengqian at School of Medicine and Health Management, Tongji Medical College, Huazhong University of Science and Technology.

This Ethics Committee is constituted and functioned in accordance with ICH-GCP, GCP in China and Declaration of Helsinki (2013).

Hui Chen	[signature]	30/12/2014
Printed Name	Signature	Date

IEC Chairperson/Designee
